# The Role of Long Non-coding RNAs in Cancer Metabolism: A Concise Review

**DOI:** 10.3389/fonc.2020.555825

**Published:** 2020-10-06

**Authors:** Soudeh Ghafouri-Fard, Hamed Shoorei, Mohammad Taheri

**Affiliations:** ^1^Department of Medical Genetics, Shahid Beheshti University of Medical Sciences, Tehran, Iran; ^2^Department of Anatomical Sciences, Faculty of Medicine, Birjand University of Medical Sciences, Birjand, Iran; ^3^Urogenital Stem Cell Research Center, Shahid Beheshti University of Medical Sciences, Tehran, Iran

**Keywords:** lncRNA, cancer metabolism, expression, biomarker, oncogene

## Abstract

Dysregulation of metabolic pathways in cancer cells is regarded as a hallmark of cancer. Identification of these abnormalities in cancer cells dates back to more than six decades, far before discovery of oncogenes and tumor suppressor genes. Based on the importance of these pathways, several researchers have aimed at modulation of these functions to intervene with the pathogenic course of cancer. Numerous genes have been shown to participate in the regulation of metabolic pathways, thus aberrant expression of these genes can be involved in the pathogenesis of cancer. The recent decade has experienced a significant attention toward the role of long non-coding RNAs (lncRNAs) in the biological functions. These transcripts regulate expression of genes at several levels, therefore influencing the activity of cancer-related pathways. Among the most affected pathways are those modulating glucose homeostasis, as well as amino acid and lipid metabolism. Moreover, critical roles of lncRNAs in regulation of mitochondrial function potentiate these transcripts as novel targets for cancer treatment. In the current review, we summarize the most recent literature regarding the role of lncRNAs in the cancer metabolism and their significance in the design of therapeutic modalities.

## Introduction

Altered metabolic pathways in cancer has been attracting researchers for more than six decades when Warburg hypothesized that the tumorigenesis process is initiated by a deficient cellular respiration due to the mitochondrial function impairment (1). This research area remarkably precedes the identification of the role of oncogenes and tumor suppressors in the carcinogenesis (2). While normal cells obtain energy principally via mitochondrial oxidative phosphorylation (3), cancer cells can fulfill the requirements of their fast and uncontrolled proliferation by excessive glycolysis and the subsequent lactic acid fermentation even in the existence of plentiful oxygen supply. This kind of aerobic glycolysis has been characterized as the Warburg effect (4). Carcinogenesis process is accompanied by the extensive synchronized activation of metabolic pathways that maintain this process by dysregulation of PI3K-AKT-mTOR signaling pathways, deficiency of tumor suppressors, and induction of oncogenes (2). The altered metabolic functions in the cancer cells have been shown to facilitate the attainment and preservation of malignant features. Since some of these characteristics have been detected rather commonly across many kinds of cancer cells, altered metabolic function is regarded as a hallmark of cancer (5). This aberrant metabolic function facilitates anabolic growth in the course of nutrient-depleted situations, catabolism to sustain cell survival for the period of nutrient insufficiency, and protection of redox homeostasis to neutralize the metabolic influences of oncogene activation, tumor suppressor deficiency or other cellular stresses (6). Such metabolic reprogramming involves several genes and molecular pathways among them are long non-coding RNAs (lncRNAs) (7). These transcripts comprise a large proportion of human transcriptome, have sizes larger than 200 nucleotides and share several features with mRNA coding genes; yet, they lack considerable open reading frames (8). Not only can they regulate cell proliferation, cell death, migration, invasion and stemness properties (9), but also they have critical roles in the regulation of cancer metabolism (7). The latter has been supported by a bunch of evidence which reports aberrant expression of metabolism-related lncRNAs in cancer cells. Moreover, functional studies have verified their roles in the context of cancer in some cases. The current review has focused on the role of lncRNAs in cancer metabolism and provides key examples in this regard. Based on the ever growing literature on this topic, this review cannot provide the exhaustive list of all related researches.

## Mechanisms of lncRNAs Functions in Regulation of Gene Expression

LncRNAs can exert their regulatory functions through different mechanisms such as modulation of chromatin structure and DNA methylation status and interacting with transcription factors and DNA motifs, thus regulating transcription of target genes. They also influence mRNA processing to affect gene expression at post-transcriptional level. Besides, their interactions with certain proteins enable them to regulate protein translation and post-translational alterations such as phosphorylation and ubiquitination (10). These transcripts can function as miRNA sponges to modulate expression of miRNA target genes or serve as precursors for miRNA or small interfering RNAs (10). LncRNAs can also modulate alternative splicing processes and consequently modulate spatial and temporal expression of genes (10).

## Oncogenic lncRNAs That Regulate Cancer Metabolism

Oncogenic lncRNAs regulate different aspects of cancer metabolism such as glutaminolysis and lipid metabolism. For instance, UCA1 has an established role in the regulation of glutamine metabolism (11). Expression of this lncRNA in bladder cancer tissues and cell lines is significantly correlated with GLS2 expression. Moreover, up-regulation of UCA1 enhances GLS2 expression and increases glutaminolysis in these cells. This function has been mediated through sponging miR-16, a miRNA that targets GLS2 (11). Besides, the oncogenic lncRNA CCAT2 has been shown to alter glutamine metabolism in colon cancer (12). Functional studies revealed the interaction between CCAT2 and CFIm complex, a protein complex that modulates the alternative splicing and the poly(A) site choosing in GLS transcript, leading to the privileged expression of the more aggressive variant GAC (12). The lncRNA PCGEM1 has been shown to enhance glucose entry in the cells to increase aerobic glycolysis, coupling with the pentose phosphate shunt to enhance lipogenesis in prostate cancer cells (12).

**Figure 1:**
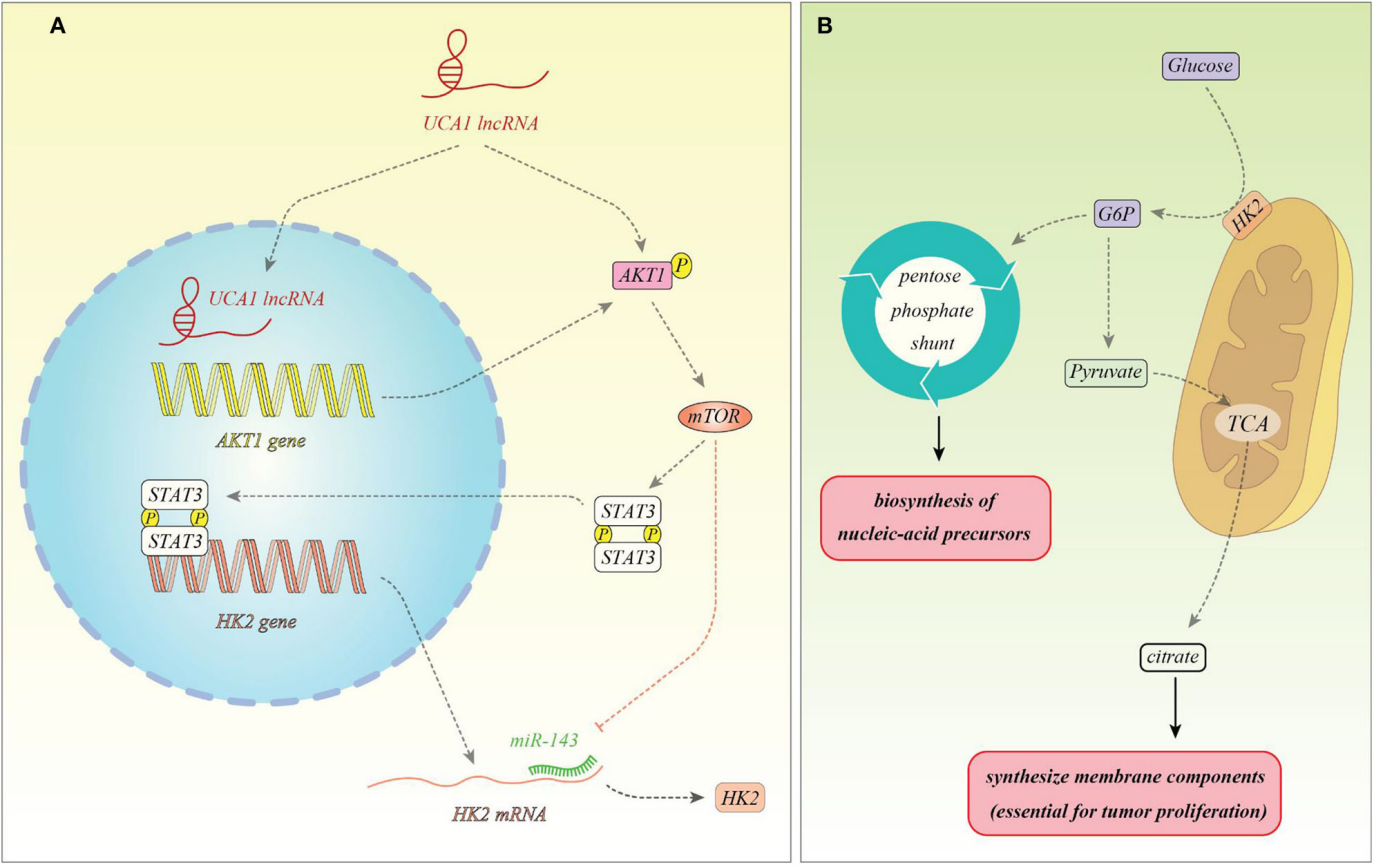
**(A)** UCA1 can enhance expression of AKT1 and activate it through phosphorylation. AKT1 activates mTOR, thus facilitating STAT3 phosphorylation and nuclear translocation. These events lead to over-expression of HK2. In addition, mTOR inhibits miR-143 which targets HK2. Therefore, mTOR has a prominent effect in enhancing HK2 expression (21). **(B)** HK2 is located in the mitochondrial membrane and facilitates conversion of glucose to glucose-6-phosphte. Glucose-6-phosphate enters the pentose phosphate pathway to produce pentose phosphate for nucleic acid synthesis which is needed for proliferation of cancer cells. In addition, glucose-6-phosphate can be converted to pyruvate to enter tri-carboxylic acid (TCA) cycle and produce citrate which is needed for the synthesis of membrane components (phospholipids and cholesterol), thus being necessary for tumor proliferation (22).

Several other up-regulated lncRNAs in the cancer cells have been shown to alter cancer metabolism. The metabolism-induced tumor activator 1 (MITA1) is an lncRNA which has been shown to be over-expressed in hepatocellular carcinoma (HCC) and participates in the metastatic potential of these cells. This lncRNA is remarkably activated by energy stress. This process is controlled by the LKB1-AMPK pathway and DNA methylation (13). Another experiment in the HCC cells has shown correlation between expressions of the lncRNA RAET1K and both HIF1A and miR-100-5p. LncRNA RAET1K has been shown to act as a molecular sponge for miR-100-5p, thus inhibiting its expression. On the other hand, HIF1A binds with the promoter region of lncRNA RAET1K to enhance its transcription. LncRNA RAET1K knock down has inhibited proliferation and invasion of HCC cells and also overturned hypoxia-induced upsurge in lactate levels and glucose uptake. Functional studies have verified the role of the HIF1A/lncRNA RAET1K/miR-100-5p axis in regulation of hypoxia-induced glycolysis in HCC cells (14). PVT1, as an up-regulated lncRNA in HCC tissues and cell lines, can directly interact with miR-150 to suppress its expression and subsequently up-regulating expression hypoxia-inducible protein 2 (HIG2) which is targeted by the miR-150. The PVT1/miR-150/HIG2 axis has been shown to regulate iron metabolism in HCC cells (15). In gastric cancer cells, LINC00152 has been shown to modulate aerobic glycolysis through modulation of miR-139-5p and PRKAA1 expressions (16). LINK-A, the upregulated lncRNA in the glioma cells, has been shown to regulate expression of lactate dehydrogenase A (LDH-A), thus enhancing glycolysis and proliferation in these cells (17). Expression of HOTAIR has been increased in hepatocellular carcinoma samples. Its expression has been enhanced under hypoxia condition. Its silencing suppressed glycolysis in these cells. Functional studies verified the role of HOTAIR as a molecular sponge for miR-130a-3p. This miRNA has been shown to inhibit expression of HIF1A. HOTAIR silencing inhibited glycolysis through modulating miR-130a-3p and HIF1A in HCC cells under hypoxic conditions (18). This lncRNA regulates cancer metabolism in pancreatic adenocarcinoma cells as well. Up-regulation of HOTAIR enhances lactate synthesis, glucose uptake and ATP synthesis. Besides, it increases HK2 expression, while HK2 up-regulation had no remarkable influence on HOTAIR expression amounts. HOTAIR has been shown to enhance cancer cell energy metabolism in this kind of cancer through increasing HK2 expression (19). UCA1 has been shown to increase mitochondrial function in bladder cancer cells. This lncRNA acts as a molecular sponge for miR-195 to control mitochondrial function through enhancing expression of ARL2. The role of UCA1 through miR-195/ARL2 axis in promotion of bladder tumor growth has been verified in animal models (20). Figure 1 shows a summary of role of UCA1 in the regulation of cancer metabolism.

Table 1 summarizes the role of oncogenic lncRNAs in the cancer metabolism in all kinds of human malignancies.

**Table 1:** The role of oncogenic lncRNAs in the cancer metabolism.

**Type of cancer**	**lncRNA**	**Numbers of clinical samples**	**Assessed cell line**	**Targets/ regulators**	**Signaling pathways**	**Function**	**Patient's prognosis**	**References**
Hepatocellular carcinoma (HCC)	MITA1	13 pairs of liver tumors and adjacent normal livers	HepG2, A549, U87, PC3, Huh7, HCCLM3, SK-Hep1, SMMC-7721, LO2, HGC27, U251	LKB1, AMPK	Slug	Energy stress through the LKB1-AMPK pathway could induce MITA1 expression. MITA1 could been induced by glucose starvation in a time-dependent manner.	–	(13)
	RAET1K	66 pairs of HCC and adjacent normal tissues	L02, HCCLM3, HepG2, huh7, Hep3B	miR-100-5p, LDHA	–	The HIF1A/lncRNA-RAET1K/miR-100-5p/LDHA axis could modulateglycolysis under hypoxia in HCC cells and affect HCC progression	–	(14)
	LINC01638	74 pairs of HCC and adjacent normal tissues	SNU-398, SNU-182	GLUT1	–	lncRNA-LINC01638 by increasing glucose uptake via targeting GLUT1 could promote cancer cell proliferation in HCC	–	(23)
	PVT1	47 pairs of liver tumors and adjacent normal livers	L-02, SK-HEP-1, Hep G2, SMMC-7721, BEL-7402, Hep3B2.1-7, QGY-7703X4	miR-150, HIG2	–	PVT1 via by regulating miR-150/HIG2 axis could modulate IRE/IRP regulatory system and cellular iron uptake/metabolism	–	(15)
	HOTAIR	38 pairs of hepatocellular carcinoma and adjacent normal tissues	HepG2, Huh7, LO2, 293T	miR-130a-3p, HIF1A, HK2		Knockdown of HOTAIR possibly via targeting miR-130a-3p/HIF1A could inhibit glycolysis in hepatocellular carcinoma cells stimulated with hypoxia	–	(18)
	HOTTIP	104 pairs of HCC and adjacent normal tissues	SMMC7721, HepG2, Hep3B	miR-192, miR-204, GLS1	–	miR-192 and miR-204 could suppress HOTTIP expression. miR-192/-204-lncRNA-HOTTIP axis via inhibiting GLS1 could interrupt HCC glutaminolysis.	Poor prognosis	(24)
	Ftx	73 pairs of HCC and adjacent normal tissues	LO2, Huh7, SMMC-7721, Bel-7402	TNF-α, leptin, PDK1, GLUT1, GLUT4	PPARγ	LncRNA-Ftx via targeting GLUTs through the PPARγ pathway could promote aerobic glycolysis in hepatocellular carcinoma.	Poor prognosis	(25)
Glioblastoma (GBM)	XIST	GSE50161 and GSE44971 microarrays	U87MG, U251, U343, Hs683, LN215, A172, HA1800	miR-126	IRS1/PI3K/Akt	Overexpression of lncRNA-XIST via miR-126/IRS1/PI3K/Akt pathway could enhance glucose metabolism in glioma. Knockdown of lncRNA-XIST could reduce GLUT1 and GLUT3 levels.	–	(26)
	UCA1	42 pairs of glioma tissues as well as the peritumoral brain edema (PTBE) tissues	U251, U87MG	miR-182, PFKFB2, CXCL14	–	LncRNA-UCA1/miR-182 axis by interacting PFKFB2 could induce a glycolytic phenotype in glioma.	–	(27)
	LINK-A	–	U87, U251, Has	LDHA	–	LncRNA-LINK-A via regulating LDHA could promote glycolysis and proliferation in GBM cells.	–	(17)
	LINC00689	56 pairs of glioma and adjacent normal tissues—GSE dataset	U87, U251, NHA, 293T	miR-338-3p, PKM2	–	LncRNA-LINC00689 via targeting miR-338-3p/PKM2 axis could promote glycolysis in glioma cells.	Poor prognosis	(28)
Breast cancer	YIYA (LINC00538)	35 pairs of breast cancer and adjacent normal tissues	MDA-MB-231, MCF7, BT474, 293T	PFKFB3, CDK6, FBXW7	–	LncRNA-YIYA could promote glycolysis in breast cancer.	Poor prognosis	(29)
	HISLA	Breast cancer samples (*n* = 453)	MDA-MB-231, MDA-MB-468, BT-474, MCF-7	GLUT1, GLUT3, HK2	–	Tumor-associated macrophages (TAMs) could enhance the aerobic glycolysis of breast cancer cells by extracellular vesicle (EV)-packaged lncRNA-HISLA. Blocking EV-transmitted lncRNA-HISLA via targeting GLUT1, GLUT3, and HK2 could inhibit the glycolysis in breast cancer cells.	Poor prognosis	(13)
	BCAR4	Breast cancer patients (*n* = 123)	MDA-MB-231, MDA-MB-468, 293T	HK2, PFKFB3	Hippo, Hedgehog	BCAR4/GLI2 by upregulating glycolytic enzymes HK2 and PFKFB3 could promote glycolysis in breast cancer cells. Overexpression of BCAR4 could increase glucose uptake and lactate production.	Poor prognosis	(30)
	SNHG7	30 pairs of breast cancer and adjacent normal tissues	MCF10A, MDA-MMB-436, HS578T, SKBR3, MDA-MB-231, MCF-7	miR-34a-5p, LDHA, c-Myc	–	c-Myc through the lncRNA-SNHG7/miR34a-5p/LDHA axis could regulate glycolysis in breast cancer cells.	–	(31)
	FGF13-AS1	30 pairs of breast cancer and adjacent normal tissues	MCF-10A, MCF-7, T47D, MDA-MB-453, MDA-MB-468, MDA-MB-231, 293T	GF13-AS1, IGF2BPs, c-Myc	–	Overexpression of lncRNA-FGF13-AS1 via FGF13-AS1/IGF2BPs/Myc feedback loop could inhibit glycolysis in breast cancer cells.	Poor prognosis	(32)
Bladder cancer (BC)	UCA1	Normal bladder tissue (*n* = 6), BC tissues (*n* = 22), and adjacent tissues (*n* = 10)	5637, UMUC2	miR-195, ARL2	–	Overexpression of lncRNA-UCA1 via downregulating miR-195 and upregulating ARL2 expression could promote mitochondrial function and ATP production of BC.	–	(20)
	UCA1	–	UMUC-2, 5637	HK2, miR-143	mTOR/STAT3	LncRNA-UCA1 via upregulating HK2 through the mTOR/STAT/miR-143 pathway could promote glycolysis in BC cells.	–	(21)
	UCA1	Normal bladder tissues (*n* = 6), adjacent cancer tissues (*n* = 10), bladder cancer tissues (*n* = 35)	UMUC2, 5637, BLS-211, BLZ-211	miR-16, GLS2	–	LncRNA-UCA1 via miR-16/GLS2 axis could promote glutamine metabolism in bladder cancer.	–	(11)
Osteosarcoma (OS)	TUG1	–	hFOB1.19, Saos-2, U2OS, HOS, MG63	HK2	–	Knockdown of lncRNA-TUG1 via targeting HK2 could inhibit glucose consumption and lactate production in osteosarcoma cells.	–	(33)
	HAND2-AS1	–	MG-63, SAOS-2, U-2OS, HOS, SW1353	FBP1, HIF1α	AKT	Knockdown of lncRNA-HAND2-AS1 via interacting with FBP1 and promoting HIF-1α could promote glucose metabolism under energy stress condition.	–	(34)
	PVT1	46 pairs of OS and adjacent normal tissues	U2OS, Saos-2, 143B, MG-63, hFOB	miR-497, HK2	–	LncRNA-PVT1 via regulating miR-497/HK2 axis could promote glycolysis and tumor progression in OS.	Poor prognosis	(35)
Endometrial carcinoma (ECa)	SNHG16	GEPIA database	HEC-1B, HEC-1A, RL95-2, AN3CA, EMC	miR-490-3p, HK2	–	TFAP2A/lncRNA-SNHG16 via targeting miR-490-3p/HK2 axis could regulate glycolysis of ECa cells.	Poor prognosis	(36)
Colon cancer (CC)	AWPPH	CC patients (29), normal controls (*n* = 42)	FHC, HT-29	GLUT-1	–	Although the glucose uptake was not directly measured, knockdown of lncRNA-AWPPH via downregulating GLUT-1 could inhibit colon cancer cell proliferation.	Poor prognosis	(37)
Lung cancer	LINC00857	35 pairs of lung cancer and adjacent normal tissues	H1229, H838, BEAS-2B	miR-1179, SPAG5	–	LncRNA-LINC00857 by targeting miR-1179/SPAG5 axis could regulate glycolysis in lung adenocarcinoma.	–	(38)
	IGFBP4-1	159 pairs of lung cancer and adjacent normal tissues	A549, PC-9, GLC-82, 16HBE, HBE-PIC, BEP-2D, BEAS-2B, 293T, L78	HK2, PDK1, LDHA	–	Overexpression of lncRNA-IGFBP4–1 via targeting HK2/PDK1/LDHA could affect energy metabolism and promote lung cancer progression.	–	(39)
Non-small cell lung cancer (NSCLC)	UCA1	–	16-HBE, A549, H1299, H522, 95D, H358	PKM2	mTOR	Knockdown of UCA1 by suppressing PKM2 through inactivation of the mTOR pathway could inhibit the glycolysis of NSCLC cells. UCA1 silencing could reduce the glucose consumption and lactate production.	–	(40)
	CRYBG3	23 clinical lung cancer tissues and 4 normal lung tissues	A549, H1299, Beas-2B	LDHA	–	lncRNA-CRYBG3 by interacting with LDHA could regulate glycolysis in lung cancer cells	–	(41)
	NORAD	80 pairs of NSCLC and adjacent normal tissues	A549, H1975, H1650, LK-2, H1299, H460, HBE	miR-136-5p, E2F1	–	LncRNA-NORAD via targeting miR-136-5p/E2F1 axis could promote glycolysis in NSCLC.	–	(42)
	BCYRN1	20 pairs of NSCLC and adjacent normal tissues	A549, H460, H1299, 16HBE	miR-149, PKM2	–	LncRNA-BCYRN1 by regulating the miR-149/PKM2 axis could promote glycolysis in NSCLC. BCYRN1/miR-149/PKM2 signaling pathway is involved in Warburg effect.	–	(43)
	LINC01123	92 pairs of NSCLC and adjacent normal tissues	A549, H1299, H1650, H1975, PC9, HBE	c-Myc, miR-199a-5p	–	LncRNA-LINC01123 via miR-199a-5p/c-Myc axis could promote aerobic glycolysis in NSCLC.	Poor prognosis	(44)
Prostate cancer (PC)	PCA3	20 pairs of PC and adjacent normal tissues	RWPE-1, C4-2, 22Rv1, LNCaP, PC3	CDK4, miR-1	–	LncRNA-PCA3 via targeting miR-1/CDK4 axis could regulate glycolysis in PC.	–	(45)
	SNHG16	Prostate carcinoma (*n* = 52) and normal prostate (*n* = 36) tissues	22Rv1, HPrEC	GLUT-1	–	Inhibition of lncRNA-SNHG16 by downregulating GLUT1 expression could reduce glucose uptake in prostate carcinoma	–	(46)
	PCGEM1	–	LNCaP, PC3, 293T, LNCaP/PCGEM1, LNCaP/shPCGEM1	c-Myc	–	lncRNA-PCGEM1 via targeting c-Myc could promote glucose uptake for aerobic glycolysis; therefore, it could regulate the metabolism of tumor	–	(47)
Colorectal cancer (CRC)	SNHG16	314 colorectal adenocarcinomas and 292 adjacent normal colon mucosa samples	Colo205, DLD1, HCT116, HCT15, HT29, LS174T, SW480, SW620, CaCo2	ASCL2, ETS2, c-Myc	Wnt	LncRNA-SNHG16 via Wnt pathway could affect some genes, such as HSD17B7 and INPP5D, involved in lipid metabolism in colorectal cancer	–	(48)
	LINRIS	118 pairs of CRC and adjacent normal tissues	CCD841, LOVO, RKO, CW2, SW1116, SW480, DLD-1, HCT116, HT29, COLO205	IGF2BP2	–	LncRNA-LINRIS via stabilizing IGF2BP2 could promote aerobic glycolysis in CLC	Poor prognosis	(49)
	GLCC1	95 pairs of CRC and adjacent normal tissues	SW1116, LoVo, SW480, Caco2, HT29, RKO, DLD-1, HCT116	c-Myc, LDHA, HSP90		LncRNA-GLCC1 via stabilizing c-Myc could promote glucose metabolism in CRC	Poor prognosis	(50)
	MAFG-AS1	52 pairs of colorectal cancer and adjacent normal tissues	HCT-116, HT29, SW480, LoVo	miR-147b, NDUFA4, PDK1, PFK1, PKM2	–	LncRNA-MAFG-AS1 by sponging miR-147b and activating NDUFA4 could promote glycolysis in colorectal cancer	–	(51)
	LINC00265	GSE21510 dataset	SW480, Caco-2, SW620, HCT116, HT29, HIEC	miR-216b-5p, TRIM44	–	LncRNA-LINC00265 via regulating miR-216b-5p/TRIM44 axis could promote glycolysis and lactate production in CRC	Poor prognosis	(52)
Cervical cancer (CC)	UCA1	–	HeLa, SiHa, HeLa-IRR, SiHa-IRR (radiation dosage: 76 Gy)	HK2, PKM, GLUT-1	–	In SiHa-IRR and HeLa-IRR cells, the expression of lncRNA-UCA1 and the activity of glycolysis are increased. LncRNA-UCA1 via the HK2/glycolytic pathway could regulate radioresistance in cervical cancer.	–	(53)
	UCA1	20 pairs of cervical cancer and adjacent normal tissues	HEC251, HEC-1B, Hela, N3CA, HEC-1A, RL95-2, Ishikawa3h12	miR-493-5p, HK2	–	LcRNA-UCA1 via targeting miR-493-5p/HK2 axis could modulate the glycolysis in cervical cancer	–	(54)
	LNMICC	211 paraffin-embedded tissues of cervical Cancer, 92 pairs of CC and adjacent normal tissues	SiHa, CaSki, ME180, MS751, HeLa, HeLa229, HLECs	miR-190, FASN, ACC1, ACOX1, CPT1A, FABP5	–	LncRNA-LNMICC could promote lymph nodes (LN) metastasis in cervical cancer via affecting fatty acid metabolism by recruiting the NPM1 to the FABP5 promoter and targeting miR190	Poor prognosis	(41)
Epithelial ovarian cancer (EOC)	LINC00092	48 pairs of serous ovarian cancer and adjacent normal tissues	SKOV-3, A2780	PFKFB2	–	The expression of lncRNA-LINC00092 is increased in A2780s ovarian cancer cell treated with recombinant CXCL14 protein. LINC00092 via targeting PFKFB2 could act in cancer-associated fibroblasts (CAF) to drive glycolysis in ovarian cancer.	Poor prognosis	(55)
	SNHG3	18 pairs of EOC and adjacent normal tissues—TCGA Data	IOSE80, SKOV3, TOV-21G, OVCAR-3	EIF4AIII, PKM, PDHB, IDH2, UQCRH, Kreb's cycle, OXPHOS	–	LncRNA-SNHG3 via targeting several pathways could regulate energy metabolism of EOC	Poor prognosis	(56)
	NRCP	Serous ovarian cancer (*n* = 29), normal ovarian (*n* = 11)	SKOV3, A2780	STAT1	–	The silencing of lncRNA-NRCP could reduce the levels of glucose-6-phosphate isomerase ALDOA and ALDOC. lncRNA-NRCP via STAT1 could promote glycolysis in ovarian cancer cells.	–	(57)
Pancreatic cancer	XLOC_006390	21 pairs of pancreatic tumors and adjacent normal tissues	CFPAC-1, BxPC-3	c-Myc, GDH1	–	LncRNA-XLOC_006390 via targeting GDH1 could promote glutamate metabolism by stabilizing c-Myc in pancreatic cancer.	–	(58)
Pancreatic ductal adenocarcinoma (PDAC)	PVT1	30 pairs of PDAC and adjacent normal tissues	HPAC, DANG, BXPC3, PANC1, ASPC-1, H6C7	miR-519d-3p, HIF-1A	–	Upregulation of lncRNA-PVT1 via regulating the miR-519d-3p/HIF-1A axis could promote glycolysis in PDAC.	Poor prognosis	(59)
	HOTAIR	Pancreatic adenocarcinoma (*n* = 78), adjacent healthy tissues (*n* = 51)	BxPC-3, Capan-2	HK2	–	Overexpression of lncRNA-HOTAIR via targeting HK2 could increase glucose uptake, lactate production, and ATP production in pancreatic adenocarcinoma	Poor prognosis	(19)
Esophageal squamous cell carcinoma (ESCC)	LOC148709	–	–	PFKFB3	–	LncRNA-LOC148709 by binding to and stabilizing PFKFB3 could play an important role in glycolytic reprogramming in ESCC	Poor prognosis	(60)
Hepatoblastoma	HR1	–	Huh7, 293T, HepG2, Hep2, HeLa, MCF7, K562, RD, THP-1, TZMBL, PANC-1	SREBP-1c	–	LncRNA-HR1 by inhibiting SREBP-1c could regulate hepatic lipid metabolism	–	(61)
	AT102202	–	HepG2	HMGCR	–	The expression of lncRNA-AT102202 is upregulated in HepG2 cells treated with epigallocatechin-3-gallate (EGCG). AT102202 via targeting HMGCR could play an important role in cholesterol metabolism.	–	(62)
Gastric cancer (GC)	MACC1-AS1	TCGA database, 123 formalin-fixed and paraffin-embedded (FFPE) GC tissue samples	AGS, GES-1, BGC803, BGC823, MKN45, SGC7901	MACC1, GLUT1, HK2, G6PD, MCT1	AMPK/Lin28	LncRNA-MACC1-AS1 via AMPK/Lin28 signaling-mediated mRNA stability of MACC1 could promote metabolic plasticity in GC cells.	Poor prognosis	(63)
	LINC00152	Pairs of GC and adjacent normal tissues	SUN16, AGS, MKN28, SGC7901, BGC823	miR-139-5p, PRKAA1	–	LncRNA-LINC00152/miR-139-5p by regulating PRKAA1 could facilitate aerobic glycolysis in GC cells.	–	(16)
	RP11-605F14.2, TBC1D3P5, BC130595, LINC00475, RP11-19P22.6, BC080653, XLOC-004923, AFAP1-AS1, EPB49, RP11-296I10.3	104 pairs of GC and adjacent normal tissues	–	–	–	Metabolic pathway-associated lncRNAs have a crucial role in the pathogenesis of GC.	–	(64)
Melanoma	H19	30 pairs of malignant melanoma and adjacent normal tissues	A375, SK-MEL-1, SK-MEL-5	miR-106a-5p, E2F3	–	LncRNA-H19 via miR-106a-5p/E2F3 axis could promote glucose metabolism in malignant melanoma.	Poor prognosis	(65)
Multiple myeloma (MM)	PDIA3P	Plasma cells derived from bone marrow of MM patients (*n* = 24) and normal healthy donors (*n* = 52)	OPM-2, U266, RPMI-8226, NCI-H929, MM.1S	c-Myc	G6PD/PPP	LncRNA-PDIA3P by interacting with c-Myc through G6PD/PPP pathway could regulate cell proliferation multiple myeloma.	Poor prognosis	(66)
Nasopharyngeal carcinoma (NPC)	ANRIL	88 pairs of NPC and adjacent normal tissues	NP69, N5-Tert, CNE2, CNE1, SUNE1, HONE1, HK1, S26, S18, 5-8F, 6-10B, HNE1	LDHA, GLUT1	mTOR	LncRNA-ANRIL via LDHA/GLUT1/mTOR pathway could promote cell glucose metabolism in NPC cells.	–	(67)
	XIST	25 pairs of NPC and adjacent normal samples	HK-1, C666-1, NP69	miR-381-3p, NEK5	–	Knockdown of XIST via downregulating NEK5 and upregulating miR-381-3p expression could inhibit hypoxia-induced glycolysis and metastasis in NPC cells.	–	(68)
Intrahepatic cholangiocarcinoma (ICC)	TUG1	102 pairs of ICC and adjacent normal tissues	HuH28, HuCCT1, RBE, HCCC- 9810, HIBEpiC	miR-145, Sirt3, GDH	–	LncRNA-TUG1 via miR-145/Sirt3/GDH axis could regulate glutamine metabolism and promote cancer progression.	Poor prognosis	(69)
Oral squamous cell carcinoma (OSCC)	ELF3-AS1	112 pairs of OSCC and adjacent normal tissues	SCC090,SCC25	GLUT1	–	LncRNA-ELF3-AS1 via positively regulating GLUT1 expression could promote glucose uptake in OSCC cells.	–	(70)
	P23154	4 pairs of OSCC and adjacent normal tissues	–	miR-378a-3p, GLUT1	–	LncRNA-P23154 by regulating GLUT1-mediated glycolysis could promote the invasion-metastasis potential of OSCC	–	(71)
Acute myeloid leukemia (AML)	UCA1	Bone marrow samples of 27 pediatric AML patients	HL60, HS-5, HL60/ADR	HK2, miR-125a	–	The expression of lncRNA-UCA1 is upregulated following ADR-based chemotherapy.UCA1 Knockdown by inhibiting glycolysis through the miR-125a/HK2 pathway could suppress the chemoresistance in pediatric AML cells.	–	(72)
	ANRIL	AML patients (*n* = 109), normal controls (*n* = 14)	MOLM-13, HL-60	LDHA, GLUT1	AdipoR1, AMPK, SIRT1	LncRNA-ANRIL via targeting LDHA/GLUT1 and through modulating the glucose metabolism pathway of AdipoR1/AMPK/SIRT1 could regulate AML development.	–	(73)
Head and neck squamous cell carcinoma (HNSCC)	HNGA1	4 pairs of HNSCC and adjacent normal tissues	–	miR-375, SCL2A1	–	LncRNA-HNGA1 via regulating miR-375/SCL2A1 could promote aerobic glycolysis in HNSCC.	–	(74)
–	NBR2	–	MDA-MB-23, 293T, 786-O, SLR20, BT549	GLUT1, AMPK, mTORC1	–	Treatment with phenformin has increased the expression of lncRNA-NBR2. Therefore, in response to phenformin treatment, lncRNA-NBR2 could regulate GLUT1 expression and glucose uptake in cancer cells.	–	(75)

## Tumor Suppressor lncRNAs That Regulate Cancer Metabolism

A number of studies have assessed the association between tumor suppressor lncRNAs and metabolic pathways. NEF as a down-regulated lncRNA in NSCLC tissues has been shown to regulate cell proliferation and glucose uptake in these cells through modulation of GLUT1 expression. Thus, this lncRNA can target glucose transportation to suppress lung tumorigenesis (76). LINC01537 as another tumor suppressor lncRNA has been demonstrated to enhance cellular sensitivity to nilotinib. This lncRNA also targets phosphodiesterase 2A (PDE2A) and enhance it expression through RNA–RNA interaction. Based on the role of PDE2A in energy metabolism, Warburg effect and mitochondrial respiration, LINC01537 has been identified as a regulator of cancer metabolism (77). The lncRNA GASL1 has been shown to enhance Bcl-2 expression, while down-regulating GLUT-1 expression. Thus, the role of this lncRNA in suppression of proliferation of prostate cancer cells has been exerted through modulation of metabolism (78). LINC01554, the down-regulated lncRNA in hepatocellular carcinoma has been shown to be suppressed by miR-365a. This lncRNA enhances the ubiquitin-mediated destruction of PKM2 and suppresses Akt/mTOR signaling pathway to stop aerobic glycolysis in hepatocellular cancer cells (79). FILNC1 has been identified as an energy stress-induced lncRNA. FILNC1 silencing in renal cancer cells lessens energy stress-induced apoptosis and considerably induces progression of this type of cancer. Notably, FILNC1 silencing increases glucose uptake and lactate synthesis via induction of c-Myc. In energy stress conditions, this lncRNA binds with AUF1, a c-Myc interacting protein. Thus, it prevents AUF1 from binding with c-Myc transcript, resulting in under-expression of c-Myc protein (80). Expression of the lncRNA HAND2-AS1 has been decreased in osteosarcoma tissues and serum samples of the affected patients compared with control samples. There was a significant association between serum levels of this lncRNA and tumor size. Notably, *in vitro* studies revealed that HAND2-AS1-silencing enhances osteosarcoma cell proliferation, upsurges glucose uptake and increases GLUT1 levels. Thus, HAND2-AS1 has a tumor suppressor role in osteosarcoma through modulating glucose metabolism (81). GATA6-AS is another tumor suppressor lncRNA that regulates expression of GLUT1. Up-regulation of this lncRNA has reduced glucose uptake and GLUT1 expression in the mantle cell lymphoma. Thus, the lncRNA GATA6-AS might suppress cancer cell proliferation through decreasing GLUT1 expression (82). The lncRNA MORT has a similar role in suppression of glucose uptake and GLUT1 expression in prostate cancer cell lines (83). In prostate cancer cells, up-regulation of GASL1 has enhanced Bcl-2 expression and decreased GLUT-1 levels (78). CASC8 is also involved in the regulation of the glycolysis in bladder cancer cells through modulating expression of the fibroblast growth factor receptor 1 (FGFR1). The interaction between this lncRNA and FGFR1 has been shown to suppress FGFR1-associated lactate dehydrogenase A phosphorylation, which decreases the lactate synthesis from pyruvate (84). Table 2 summarizes the role of tumor suppressor lncRNAs in the cancer metabolism.

**Table 2:** The role of tumor suppressor lncRNAs in the cancer metabolism.

**Type of cancer**	**lncRNA**	**Numbers of clinical samples**	**Assessed cell line**	**Targets/ regulators**	**Signaling pathways**	**Function**	**Patient's prognosis**	**References**
Non-small cell lung cancer (NSCLC)	NEF	33 pairs of NSCLC and adjacent normal tissues	NCI-H23, NCI-H522, NCI-H520, NCI-H2170	GLUT1	–	Overexpression of lncRNA-NEF via downregulating GLUT1 expression could inhibit glucose uptake in NSCLC cells	Poor prognosis	(76)
Lung cancer	LINC01537	243 pairs of cancerous and corresponding non-cancer lung tissues	A549, PC-9, 293T	PDE2A, GLUT1	–	Overexpression of lncRNA-LINC01537 via targeting PDE2A could attenuate the Warburg effect and mitochondrial respiration. Therefore, it is involved in energy metabolism	–	(77)
Hepatocellular carcinoma (HCC)	LINC01554	167 pairs of HCC and adjacent normal tissues	MIHA, BEL7402, QGY7701, QGY7703, SMMC7721, PLC8024, HepG2, Huh7, Hep3B	miR-365a, PKM2	Akt/mTOR	LncRNA-LINC01554-mediated glucose metabolism reprogramming via downregulating PKM2 expression and inhibiting Akt/mTOR signaling pathway could suppress tumorigenicity in HCC.	Poor prognosis	(79)
Renal cancer	FILNC1	23 pairs of ccRCC and normal kidney samples	293T, RCC4, 786-O, 769P, SLR20, UMRC2	AUF1	–	LncRNA-FILNC1 deficiency via targeting AUF1 could increase glucose uptake and lactate production in renal tumor.	Poor prognosis	(80)
Osteosarcoma	HAND2-AS1	Osteosarcoma patients (*n* = 48), normal controls (*n* = 44)	MG-63, SAOS-2, hFOB-2	GLUT1	–	Knockdown of lncRNA-HAND2-AS1 via upregulating GLUT1 expression could promote glucose uptake in osteosarcoma.	–	(81)
Mantle cell lymphoma (MCL)	GATA6-AS	Plasma samples of patients with MCL (*n* = 47) and healthy controls (*n* = 42)	JVM-2, Z-138	GLUT1	–	Overexpression of lncRNA-GATA6-AS by downregulating GLUT1 expression could inhibit glucose uptake in mantle cell lymphoma.	–	(82)
Bladder cancer	CASC8	50 pairs of bladder cancer and adjacent normal tissues	SW780, J82, UMUC3, T24, 5637	FGFR1	–	Overexpression of CASC8 through interacting with FGFR1 and inhibiting FGFR1-mediated LDHA phosphorylation could suppress glycolysis in bladder cancer cell.	–	(84)
Prostate carcinoma (PC)	GASL1	66 pairs of PC and adjacent normal tissues	HprEC, 22Rv1, DU145	GLUT-1, Bcl-2, Bax	–	GASL1 via targeting GLUT-1, which has a major role in glucose metabolism, could promote the expression of apoptosis-associated proteins in PC cells; hence, could inhibit the growth.	Poor prognosis	(78)
	MORT	60 pairs of PC and adjacent normal tissues	22Rv1	GLUT-1	–	lncRNA-MORT by inhibiting glucose uptake via inactivating GLUT-1 expression could suppress tumor cell proliferation in PC.	–	(83)
Gastric cancer	TUG1, RP11-555H23.1, RP1-257I20.13, UGP2, GCSHP3, XLOC-000889	104 pairs of gastric carcinoma and adjacent normal tissues	–	–	–	Metabolic pathway-associated lncRNAs have a crucial role in gastric cancer.	–	(64)
	TOPORS-AS1	103 pairs of GC and adjacent normal tissues	–	NDUFB6	–	The metabolism-associated lncRNAs have important roles on metabolism of cancers. TOPORS-AS1 via targeting NDUFB6 may affect glucose metabolism in gastric cancer cells.	-	(85)
Several human cancers	NBR2	–	MDA-MB-23, 293T, HeLa, A549, 786-O, DU145, MCF-7, BT-549, SLR20	LKB1	AMPK	NBR2 via LKB1–AMPK pathway could engage a metabolic checkpoint under energy stress.	Poor prognosis	(86)
	EPB41L4A-AS1	TCGA and GEO datasets	HeLa, HepG2	HDAC2, HIF-1α, VDAC1, VHL	–	lncRNA-EPB41L4A-AS1 via mediating nucleolar translocation of HDAC2 could regulate glycolysis and glutaminolysis in cancer.	–	(87)
Colorectal cancer	MEG3	80 colorectal cancer tissue samples and adjacent normal mucosal samples	DLD-1, RKO	c-Myc	–	Vitamin D-activated lncRNA-MEG3 via degrading c-Myc could suppress aerobic glycolysis of colorectal cancer cells.	Poor prognosis	(88)

## Significance of Metabolism-Related lncRNAs in Cancer Diagnosis and Prognosis

Consistent with the crucial roles of metabolism-related lncRNAs in the evolution of human cancers, dysregulation of these lncRNAs have been associated with patients' outcome. Moreover, transcript levels of them have been exploited as diagnostic markers in diverse cancers. For instance, serum levels of the lncRNA AWPPH have been elevated in patients with colon cancer compared with normal subjects. Receiver operating characteristic (ROC) curve analysis has shown the appropriateness of serum levels of this lncRNA for diagnosis of colon cancer with diagnostic power of 0.84 (37). Serum levels of the lncRNA NEF have been shown to have diagnostic power of 0.94 for NSCLC. Moreover, the overall survival rate of patients with elevated serum levels of this lncRNA was remarkably better compared with those having low level of this lncRNA. Taken together, serum concentrations of NEF might be considered as diagnostic and prognostic markers for this kind of cancer (76). In patients with cholangiocarcinoma, Kaplan-Meier survival analysis has demonstrated decreased overall survival (OS) and disease-free survival (DFS) in patients with high levels of TUG1 expression. Univariate analysis has also verified the effect of TUG1 expression levels in determination of OS and DFS (69). Several other lncRNAs that regulate cancer metabolism have been identified as diagnostic/prognostic markers in cancer. Table 3 summarizes the results of studies which reported diagnostic/prognostic significance of these lncRNAs.

**Table 3:** The role of metabolism-related lncRNAs in cancer diagnosis and prognosis (DFS, disease free survival; OS, overall survival; PFS, progression free survival).

**Sample number**	**AUC**	**Sensitivity**	**Specificity**	**Kaplan-Meier analysis**	**Univariate cox regression**	**Multivariate cox regression**	**References**
Colon cancer patients (29), normal controls (*n* = 42)	0.84 for AWPPH	–	–	–	–	–	(37)
33 pairs of NSCLC and adjacent normal tissues	0.94	–	–	Patients with a high-level lncRNA-NEF had a higher rate of OS.	–	–	(76)
Osteosarcoma patients (*n* = 48), normal controls (*n* = 44)	0.86	–	–	–	–	There was a significant correlation between tumor size and serum levels of HAND2-AS1	(81)
Breast cancer samples (*n* = 453)	–	–	–	Patients with a high level lncRNA-HISLA had lower rate of OS.	–	–	(13)
80 colorectal cancer tissue samples and adjacent normal mucosal samples	–	–	–	Patients with a low-level lncRNA-MEG3 had a lower rate of OS.	–	–	(88)
30 pairs of pancreatic cancer and adjacent normal tissues	–	–	–	Patients with a high-level lncRNA-PVT1 had a lower rate of OS.	–	–	(59)
104 pairs of gastric carcinoma and adjacent normal tissues	0.65 for lncRNA- RP11-555H23.1	81% for lncRNA- RP11-555H23.1	62% for lncRNA- RP11-555H23.1	–	–	RP11-555H23.1 expression was significantly correlated with TNM stage	(64)
104 pairs of HCC and adjacent normal tissues	–	–	–	Patients with a high-level lncRNA-HOTTIP had a lower rate of OS.	–	–	(24)
18 pairs of ovarian cancer and adjacent normal tissues—TCGA Data	–	–	–	Patients with a high-level lncRNA-SNHG3 had a lower rate of OS.	–	–	(56)
56 pairs of glioma and adjacent normal tissues—GSE dataset	–	–	–	Patients with a high-level lncRNA-LINC00689 had a lower rate of OS.	–	–	(28)
30 pairs of malignant melanoma and adjacent normal tissues	–	–	–	Patients with a high-level lncRNA-H19 had a lower rate of OS.	–	–	(65)
Pancreatic adenocarcinoma (*n* = 78), adjacent healthy tissues (*n* = 51)	–	–	–	Patients with a high level lncRNA-HOTAIR had lower rate of OS.	–	–	(19)
66 pairs of prostate cancer and adjacent normal tissues	0.9076 for tissue, 0.8811 for serum	–	–	Patients with a low-level lncRNA-GASL1 had a lower rate of OS.	–	Expression levels of GASL1 were significantly associated with tumor size.	(78)
88 pairs of NPC and adjacent normal tissues	–	–	–	–	–	ANRIL expression could serve as an independent predictor of disease-free survival and overall survival	(67)
102 pairs of ICC and adjacent normal tissues	–	–	–	–	No association was observed between TUG1 expression and age, sex, and tumor size	TUG1 expression was associated with tumor stage, intrahepatic metastasis, lymph node metastasis, and perineural invasion	(69)
Serous ovarian cancer (*n* = 29), normal ovarian (*n* = 11)	–	–	–	Patients with a high-level lncRNA-NRCP had lower rate of OS.	–	–	(57)
118 pairs of CRC and adjacent normal tissues	–	–	–	Patients with a high-level lncRNA-LINRIS had a lower rate of OS.	–	–	(49)
92 pairs of NSCLC and adjacent normal tissues			–	Patients with a high-level lncRNA-LINC01123 had a lower rate of OS.	–	–	(44)
211 paraffin-embedded tissues of the cervical Cancer, 92 pairs of CC and adjacent normal tissues	–	–	–	Patients with a high-level lncRNA-LNMICC had lower rates of OS and DFS.	–	A higher LNMICC expression was correlated with tumor size, lymph node metastasis, lymphovascular space invasion, stromal invasion, recurrence, and vital status	(41)
23 pairs of ccRCC and normal kidney samples	–	–	–	Patients with a high-level lncRNA-FILNC1 had a lower rate of OS.	–	–	(80)
95 pairs of CRC and adjacent normal tissues	–	–	–	–	lncRNA-GLCC1 expression was an independent predictor of CRC aggressiveness	the lncRNA-GLCC1 expression is associated with tumor size	(50)
46 pairs of OS and adjacent normal tissues	–	–	–	Patients with a high-level lncRNA-PVT1 had a lower rate of OS.	–	–	(35)
GSE21510 dataset	–	–	–	Patients with a high level lncRNA-LINC00265 had lower rate of OS.	–	–	(52)
167 pairs of HCC and adjacent normal tissues	–	–	–	Patients with a low-level lncRNA-LINC01554 had a lower rate of OS.	–	LINC01554 was associated with tumor invasion, tumor size, tumor staging in HCC patients.	(79)
TCGA database, 123 formalin-fixed and paraffin-embedded (FFPE) GC tissue samples	–	–	–	Patients with a high-level lncRNA-MACC1-AS1 had lower rates of OS and DFS.	–	MACC1-AS1 and TNM stage were independent prognostic factors in GC patients.	(63)
48 pairs of serous ovarian cancer and adjacent normal tissues	–	–	–	Patients with a high-level lncRNA-LINC00092 had lower rates of OS and PFS.	–	–	(55)
Plasma cells derived from bone marrow of MM patients (*n* = 24) and normal healthy donors (*n* = 52)	–	–	–	Patients with a high-level lncRNA-PDIA3P had lower rate of OS.	–	–	(66)

## Discussion

The carcinogenesis process is associated with high glucose uptake, lactate over-production, aerobic glycolysis as well as glutamine and lipid metabolism (89). The above-mentioned data support the role of lncRNAs in these metabolic pathways in the context of cancer. Notably, HCC has been the most investigated cancer type regarding the role of lncRNAs in the metabolic pathways. Apart from the function of lncRNAs in this regard, metabolic changes have been previously recognized to evidently distinguish HCC tumors. Several clinical parameters that are presently utilized to evaluate liver functions reveal alterations in both enzyme activity and metabolites. Actually, alterations in glucose and acetate consumption are regarded as effective clinical means for classification of patients with HCC. Besides, elevated serum lactate can differentiate HCC from healthy individuals, and serum lactate dehydrogenase is applied as a determinant of prognosis of HCC patients under therapeutic regimens (90). Thus, it is not surprising that the role of lncRNAs has been vastly assessed in this context. The underlying mechanism of participation of lncRNAs in the regulation of metabolic pathways has been clarified in several cases. Glucose transporters (GLUTs) as important modulators of glucose utilization which are commonly dysregulated in cancer (91), have been shown to be targeted by several lncRNAs such as LINC01638, Ftx, XIST, YIYA (LINC00538), HISLA, AWPPH, and UCA1. Most notably, several lncRNA/ miRNA/mRNA comprising axes have been shown to modulate cancer metabolism. Therefore, the complex interactions between these trios should be considered in the design of any therapeutic option. Moreover, numerous lncRNAs have direct or indirect interactions with the well-known oncogene c-Myc. This oncogene is an important modulator of pathways that regulate metabolism of glucose, glutamine and lipid in cancer (92). Thus, all of these lncRNAs are putative regulators of different aspects of cancer metabolism.

Several oncogenic lncRNAs mainly exert their effects through modulation of these pathways. Thus, modulation of expression of these lncRNAs through application of antisense oligonucleotides or CRISPR/Cas9-based modalities can be regarded as a therapeutic option in cancer. Yet, the main obstacles in this regard are their off-target effects or unstable efficiency resulting from the space-time related features of lncRNAs (93). Small interfering (si)RNA-mediated silencing of oncogenic lncRNAs has been hampered by unavailability of efficient delivering systems. Yet, this such hurdle has been rather solved by the advent of biocompatible nanoparticle delivery systems (86).

The relevance of metabolism-associated lncRNAs in the treatment of cancer has been highlighted by a number of studies. For instance, the lncRNA-UCA1 has been shown to modulate radioresistance in cervical cancer cell through the HK2/glycolytic pathway (53). The same lncRNA has been shown to be upregulated in AML patients after Adriamycin (ADR)-based chemotherapy. UCA1 silencing has enhanced the cytotoxic effect of this chemotherapeutic agent and suppressed the HIF-1α-associated glycolysis in ADR-resistant AML cells. Based on these results, UCA1 has been shown to exert a positive role in conquering the chemoresistance in pediatric AML patients (72).

Several lncRNAs such as NEF, HISLA, MEG3, PVT1, HOTTIP, SNHG3, LINC00689, H19, HOTAIR, GASL1, NRCP, LINRIS, and FILNC1 have been identified as predictive markers for OS or DFS of cancer patients. The potential of a number of lncRNAs including AWPPH, NEF, HAND2-AS1, lncRNA- RP11-555H23.1, and GASL1 as diagnostic markers in cancer patients has also been verified. These data suggest the importance of these lncRNAs in diverse aspects of cancer biology.

Taken together, regulation of cancer metabolism is a critical role of lncRNAs which has been shown by several *in vitro* investigations and a number of clinical studies. Thus, these transcripts are putative therapeutic targets in cancer. The importance of this function of lncRNAs is further highlighted by the eminent role of tumor microenvironment in the evolution of cancer and the ubiquitous dysregulation of metabolism in different cancer types. Thus, therapeutic targeting of these lncRNAs can be applied in diverse cancer types.

## Author Contributions

SG-F and MT wrote the draft and revised it. HS collected the required information and data. All authors contributed equally and fully aware of submission.

## Conflict of Interest

The authors declare that the research was conducted in the absence of any commercial or financial relationships that could be construed as a potential conflict of interest.
